# Editorial: Impact and mitigation of abiotic stress in cereals

**DOI:** 10.3389/fpls.2024.1374631

**Published:** 2024-02-07

**Authors:** Peter Christian Morris, Moshe Reuveni, Rodomiro Ortiz

**Affiliations:** ^1^Institute of Life and Earth Sciences, Heriot-Watt University, Edinburgh, United Kingdom; ^2^Institute of Plant Sciences, Agricultural Research Organization, Volcani Center, Rishon LeZion, Israel; ^3^Department of Plant Breeding, Swedish University of Agricultural Sciences, Uppsala, Sweden

**Keywords:** abiotic stress, drought, heat, salinity, cereals

Humanity has entered an unprecedented era of climatic change with the past decade showing the fastest rise of global temperature on record and 2023 being the warmest year in 174 years of observation (Intergovernmental Panel on Climate Change (IPCC), 2023). These facts bring into sharp focus the impact of abiotic stresses on food supplies. Human sustenance depends on daily caloric intake mainly by means of the consumption of proteins, fats and carbohydrates from various cellular sources. A single human needs to consume at least 2000 calories in food every day or about 730,000 a year (Dietary guidlines.gov, 2020). Current global food production (including that destined for animal feed) is about 2,900 calories per person per day (FAO, 2022). However, the projected increase in world population is to around 10 billion people in the next twenty to thirty years, and feeding the increasing populace is a primary concern for many governments and policymakers in agriculture. Food production for the expected 10 billion population by the year 2050 needs to supply at least 20^12^ calories a day. Cereals in particular form a major component of global human diets with nearly half of calories directly derived from the seeds of these cultivated grasses (Jones, 2023), and even more from the use of cereals as animal feed and in the beverage industries. Abiotic stresses (for example, heat, drought) are exacerbated by climate change and have a negative impact on cereal crop quality and yield. Climate change is currently thought to be a major contributor to reductions in cereal yields worldwide (Wang et al., 2018). In order to provide the estimated 70 to 100% increase in cereals to feed an expanding world population (Godfray et al., 2010), it is imperative for plant scientists to better understand all aspects of abiotic stress on cereal crops, from perception and signal transduction through plant strategies for stress avoidance and mitigation to consider genetic resources and agronomic solutions to enhance production under stressful conditions (Sato et al., 2024).

The major abiotic factors that impact plant growth are heat, drought and salinity, which have distinct but overlapping effects on plant physiology but other abiotic factors (for example, anoxia and cold) are also of importance. In this Research Topic we explore some current advances in our understanding of how cereal crop plants can perceive, and how they respond to abiotic stresses, including both stress avoidance and stress tolerance. Strategies for mitigation through agronomic practice, and also longer-term breeding goals are considered.

The study of abiotic stress in crop plants covers a gamut of approaches, ranging from environmental research through physiological investigations and molecular and genetic aspects of plant responses to stresses. The Research Topic presented here reflect these different aspects of the science. The paper by Bal et al. analyses the impact of large-scale weather patterns across different environments on the yield of one rice cultivar over a four-year period. The results not only show how the climate (in particular, temperature and rainfall) impacts upon yield, but shows how the local environment at particular growth stages such as seedling establishment or seed filling can affect this crop.

The local crop environment can cause stress to plants through direct anthropogenic factors such as fertiliser regimes, and the paper by Marti-Jerez et al. has used remote satellite sensing together with ground-based assessments in a six-year longitudinal study to monitor the effect of mineral and organic fertilisers on rice performance in the field. This work shows that under the conditions used, mineral fertilisers gave a slight overall yield advantage, which was related to nitrogen deficiencies at later stages of growth when the organic fertilisers were used; however, organic fertilisers have the advantage of enhancing the available soil phosphorus and potassium levels. The satellite-based monitoring system showed promise for the detection of early signs of nutrient deficiencies which could then be addressed in a targeted manner.

The direct physiological effects of drought on crops have been well investigated by numerous studies, in particular crop water content (Ievinsh, 2023). The parameters defining water content have been more closely examined in the paper by Yan et al. who investigated the allocation of water and biomass within maize during periods of abiotic stress. The authors found differential retention of water under drought, which was reduced in roots and stems, and also of biomass which increased in roots and seeds. This work shows that simplistic whole-plant models of drought response are insufficient for a proper understanding of drought stress in crops, and that a more nuanced approach is required for a full understanding of water content fluctuations.

An understanding of the molecular mechanisms underlying stress tolerance is of critical importance when considering long term breeding strategies for stress resilient crops. The paper by Xing et al. investigates some fundamental mechanisms that regulate primary root growth under salt stress in maize. They find that the microRNA ZmmiR169q is a negative regulator of the transcription factor ZmNF-YA8. Salt stress reduces *ZmmiR169q* expression and thus enhances *ZmNF-YA8*, which together with input from ethylene signalling, leads to the upregulation of *ASA1* and *ASA2*, involved in tryptophan and auxin synthesis. High levels of auxin lead then to inhibition of primary root growth and arrested growth.

Bringing together environmental, physiological, and molecular research on stress responses of crops opens up the possibility of engineering metabolic pathways for stress tolerance. This is explored in the review paper by Liu et al. Their analysis suggests that selection for or modification of single gene traits in plants is unlikely to yield large improvements in stress tolerance. What may be more fruitful is an approach that targets multiple genes or loci that modify metabolic pathways with multiple outputs such as starch metabolism, phenylpropanoid metabolism or phytohormone signalling.

Taken together, this Research Topic illustrates the range and impact that plant scientists are having on addressing one of the most important problems to face the world in modern time; i.e., how to continue to feed the worlds population in a sustainable manner.

## Author contributions

PM: Writing original draft, Writing review & editing. MR: Writing review & editing. RO: Writing review & editing.
